# Czechoslovakian Wolfdog Genomic Divergence from Its Ancestors *Canis lupus*, German Shepherd Dog, and Different Sheepdogs of European Origin

**DOI:** 10.3390/genes12060832

**Published:** 2021-05-28

**Authors:** Nina Moravčíková, Radovan Kasarda, Radoslav Židek, Luboš Vostrý, Hana Vostrá-Vydrová, Jakub Vašek, Daniela Čílová

**Affiliations:** 1 Department of Animal Genetics and Breeding Biology, Faculty of Agrobiology and Food Resources, Slovak University of Agriculture in Nitra, Tr. A. Hlinku 2, 94976 Nitra, Slovakia; nina.moravcikova@uniag.sk; 2 Department of Food Hygiene and Safety, Faculty of Biotechnology and Food Sciences, Slovak University of Agriculture in Nitra, Tr. A. Hlinku 2, 94976 Nitra, Slovakia; radoslav.zidek@uniag.sk; 3 NU3gen, Pažite 145/7, 010 09 Žilina, Slovakia; 4 Department of Genetics and Breeding, Faculty of Agrobiology, Food and Natural Resources, Czech University of Life Sciences Prague, Kamycka 129, 165 00 Prague, Czech Republic; vostry@af.czu.cz (L.V.); vasek@af.czu.cz (J.V.); cilova@af.czu.cz (D.Č.); 5 Department of Ethology and Companion Animal Science, Faculty of Agrobiology, Food and Natural Resources, Czech University of Life Sciences Prague, Kamycka 129, 165 00 Prague, Czech Republic; vostrah@af.czu.cz

**Keywords:** behaviour, dogs, genomic diversity, morphological traits, protein-coding genes, selection events

## Abstract

This study focused on the genomic differences between the Czechoslovakian wolfdog (CWD) and its ancestors, the Grey wolf (GW) and German Shepherd dog. The Saarloos wolfdog and Belgian Shepherd dog were also included to study the level of GW genetics retained in the genome of domesticated breeds. The dataset consisted of 131 animals and 143,593 single nucleotide polymorphisms (SNPs). The effects of demographic history on the overall genome structure were determined by screening the distribution of the homozygous segments. The genetic variance distributed within and between groups was quantified by genetic distances, the *F_ST_* index, and discriminant analysis of principal components. Fine-scale population stratification due to specific morphological and behavioural traits was assessed by principal component and factorial analyses. In the CWD, a demographic history effect was manifested mainly in a high genome-wide proportion of short homozygous segments corresponding to a historical load of inbreeding derived from founders. The observed proportion of long homozygous segments indicated that the inbreeding events shaped the CWD genome relatively recently compared to other groups. Even if there was a significant increase in genetic similarity among wolf-like breeds, they were genetically separated from each other. Moreover, this study showed that the CWD genome carries private alleles that are not found in either wolves or other dog breeds analysed in this study.

## 1. Introduction

Dogs (*Canis lupus familiaris*) originating from a substantial number of grey wolves (*Canis lupus*) express different breed-specific phenotypic traits. Even if six dog breeds are generally recognised as the product of grey wolves crossing with domestic dogs, only the Czechoslovakian Wolfdog (CWD) and Saarloos Wolfdog (SWD) are accepted by the Federation Cynologique Internationale (FCI) as wolf hybrid breeds. They were created at independent times from the crossing of the German Shepherd dog (GSD) with grey wolves. These breeds belong to the FCI Group 1, which contains sheepdogs and cattle dogs (except Swiss cattle dogs), including the Belgian Shepherd dog (BSD). Although the BSD is distantly related to the CWD, SWD, and GSD, it has been bred for similar functional purposes.

The CWD was first recognised as a breed on the national level in 1989 and was registered by the FCI in 1999 (FCI Standard No. 332). In the beginning, the breeding goal was to create a working dog based on the hybridisation of the Carpathian grey wolf (GW) and GSD for military purposes to patrol the country’s borders. Later, the working abilities of the CWD were depressed in the breeding goal (except endurance), focusing strictly on wolf-like morphology and wolf-like coat texture, coat colours, and mask. The CWD is recognised as a temperate, persistent, and trainable dog breed. Thus, the immediate breeding goal of the wolf-like phenotype and dog-like behaviour created, in the CWD genome, a unique set of haplotypes compared to other breeds. On the other hand, the relatively low number of founders resulted in the fact that the CWD genome carries long runs of homozygosity (ROH) islands, leading to much higher inbreeding than the pedigree estimates [1]. Currently, the CWD is recognised as one of the most popular wolf-like phenotype dog breeds in the world accepted by the FCI.

The SWD breeding started in the Netherlands in 1932. This breed was accepted by the FCI on a definitive basis in 1981 under Standard No. 311. Similar to the CWD, the SWD was created by crossing a GSD with a wolf originating from the Siberian branch of the European type. The SWD is a strongly built breed whose outer appearance (body build, movement, and coat colour) is reminiscent of a wolf, similar to the CWD. Coat colours range from light to dark shaded black-tipped game-colour (boar and hare), called wolf-grey, and light to dark shaded brown-tipped game-colour. Typical wolf-markings range from light creamy white to white. The reserved and wolf-like manner to avoid unknown situations is typical for the Saarloos wolfdog [2].

The breeding club of GSDs was established in 1899. The GSD was accepted on a definitive basis by the FCI in 1955 (FCI Standard No. 166). The breeding goal is to breed a well-balanced, self-assured, good-natured, instinctive guard, protection, service and herding dog. Its origin traces back to the central and southern German breeds of herding dogs. Compared to CWDs and SWDs, the German Shepherd dog’s standard colour is black with reddish-brown, brown and yellow-to-light grey markings; the white colour is not allowed. The undercoat of GSDs has a light-greyish tone. Thus, different selection footprints in genomic regions concerning coat colour among the CWD, SWD, and GSD can be expected. However, an extensive genetic study mapping the GSD genomic diversity was not performed until now. Only several studies have assessed the level of homozygosity or inbreeding in the GSD genome as a part of autosomal inherited disease research [3] or population structure analysis [4].

The BSD, representative of sheepdogs from FCI Group 1 (FCI Standard 15, first registered on the definitive basis in 1956), was officially born between 1891 to 1897. The type and temperament of the BSD were established in 1910. It is recognised as a watchful and active, ready-to-action dog with innate skills of guarding flocks and property, as well as a defence and service dog. A previous study on assessing microsatellite markers of BSDs revealed a decrease in genetic diversity and the presence of bottlenecks without a strong effect on the BSD gene pool [5].

When considering any dog breed’s genetic makeup, repeated mating of close relatives to fix specific traits of breeds can be found. From a genomic perspective, such a mating resulted in offsprings with long ROH segments spread across the genome. Most of them have shared history through close relatives, which led to the segregation of often rare recessive or partially recessive genetic variants producing in the homozygous state a deleterious phenotype [6]. Thus, the identification of long ROH segments is of eminent interest in livestock and companion species [7,8] to analyse the effect of selective breeding on genome composition and prevent deleterious variants from acting, especially in small genetically limited populations. As a result of selective breeding, small populations lose their genetic diversity and become endangered by extinction much faster than large populations [9]. The long ROH segments were also found in the wild species’ genome, including wolves [10,11].

During domestication and the subsequent development of specialised dog breeds, a significant selection pressure acted mainly in genomic regions controlling preferred morphological (e.g., coat colour) and behavioural traits. Previous studies confirmed that the genetic and phenotypic assessment of coat colour mutations could contribute to investigating the origin and dynamics of a functional polymorphism in hybrid wolf populations and developing appropriate guidelines to contrast hybridisation with their domesticated relatives [12]. Dreger and Schmutz [13] studied specific genetic variants controlling the agouti pigment expression responsible for many coat colour patterns, such as black-and-tan, saddle tan, wolf sable, or fawn. The black colour of wolves as evidence of the admixture with domestic dogs was suggested by Randi et al. [14], Caniglia et al. [1], Saleh et al. [15], and Schweizer et al. [16]. Kerns et al. [17] identified a genetic variant in the agouti gene responsible for the uniform black coat colour in GSDs. Monteagudo et al. [18] described the association of the tyrosinase-related protein 1 gene (*TYRP1*) polymorphism with coat colour variation patterns in GSDs. Miluchová et al. [19] and Moravčíková et al. [20] studied the polymorphism in the melanophilin (*MLPH*) gene responsible for melanin transport from the skin to the hair follicle in association with slow hair growth (BHFD) or even the fall-down of hair and the presence of alopecia (CDA). In many different species and breeds, the *KIT* gene was identified as a candidate gene responsible for specific white coat colour patterns [8].

Except for coat colour variation, several studies infer differences in behaviour among conventional breeds, revealing that herding dogs are more trainable than hunting, sporting, pleasure, or companion (non-sporting) breeds [21]. The inheritance of breed-specific temperament traits irrespective of the owner’s demography or living environment was confirmed by Takeuchi and Mori [22], Kasarda et al. [23], Persson et al. [24], and vonHoldt et al. [25]. Moreover, Takeuchi et al. [26] found several polymorphic sites connected to the success of dogs in becoming qualified as guide dogs. On the other hand, polymorphisms associated with breed-specific traits, such as obedience and aggression, are still under research [27,28].

This study aimed to quantify genomic differences between the Czechoslovakian Wolfdog and its ancestors, the Grey wolf (Carpathian) and German Shepherd dog. In addition, two other sheepdogs were analysed as representatives of the same FCI section to compare their genome’s composition and study the level of Grey wolf genetics retained mainly in the CWD gene pool. The potential impact of selective breeding for specific traits of interest on their genome composition was estimated concerning polymorphisms in genes related to aerobic trainability of the organism, behaviour and motivation, coat colour, strength, and endurance.

## 2. Materials and Methods

### 2.1. Data Source

The dataset consisted of genotyping information covering the canine autosomal genome of four breeds (Belgian Shepherd dog—BSD, Czechoslovakian Wolfdog—CWD, German Shepherd dog—GSD, Saarloos Wolfdog—SWD) and the free-range Grey wolf (GW) as a common ancestor of each of them. Genome-wide data were obtained for a total of 133 animals via high-density canine BeadChip. As the dataset consisted of new and previously published data (Table 1), the map file of common autosomal single nucleotide polymorphisms (SNPs) across them was produced to avoid genome-wide screening with inconsistent information in the subsequent steps of analysis. Overall, 153,733 SNP markers were found to be common across groups. Three animals and 10,140 SNPs were pruned out due to a low genotyping rate (threshold value set to 10% of missing data). The basic manipulation and pruning of SNP data were carried out using PLINK1.9 [29]. The final dataset consisted of 131 animals and 143,593 SNPs covering 21,995.34 Mbp of the genome.

### 2.2. Effect of Selection on the Genome-Wide ROH Distribution

The effects of selective breeding in dogs and natural selection in Grey wolves on their genome composition were tested by the screening of ROH with various lengths (0–2, 2–4, 4–8, 8–16, and >16 Mbp) to distinguish between genetic information inherited from different ancestor generations. The ROH were defined as genomic segments with a minimum length of 500 kb [6] containing at least 65 consecutive homozygous SNP genotypes no more than 1000 kb apart. The heterozygous or missing genotypes in ROH windows were not allowed. Each ROH had to have at least one SNP per 5000 kb on average. The minimum number of SNPs in the ROH window was calculated following Lencz et al. [31] as follows:

$$l=\frac{log_{e}\alpha /n_{s}n_{i}}{log_{e}\left(1-\bar{het}\right)},$$

where *α* is the proportion of false-positive ROH segments (set to 0.05; Mastrangelo et al. [32]), *n_s_* is the total SNP number, *n_i_* is the number of analysed animals, and $\bar{het}$ expresses the overall average heterozygosity across SNPs and animals. The proportion of ROH with various lengths in the genome was scanned using the detectRUNS R package [33] separately for each analysed group. The trend of the relative mating proportion in the individual’s genome across different ancestor generations expressed as genomic inbreeding (*F_ROH_*) was then calculated by dividing the sum of the ROH length (kb) in the particular length class by the total length of the autosomal genome (kb) covered by the SNPs. Five inbreeding coefficients for each individual were calculated.

### 2.3. Population Structure of the Breeds

The systematic differences in allele frequencies among tested groups as a consequence of nonrandom mating between animals related to selective breeding for traits of interest were tested by several methodological approaches. The overall degree of population stratification was assessed by calculating Nei’s genetic distances (*D_a_*) between animals and the pair-wise Wright’s *F_ST_* index at the group level using the StAMPP R package [34]. 

The discriminant analysis of principal components (DAPC) was used to quantify the variance distributed within and between clusters of genetically related animals. The DAPC analysis was based on the pre-defined groups reflecting the origin of analysed animals. The DAPC analysis allowed for the transformation of the input genotype data using principal component analysis (PCA) to uncorrelated variables describing the target proportion of variance in the dataset. In the subsequent discriminant analysis (DA), the uncorrelated variables were used to maximise inter-group variance estimation and obtain discriminant functions representing a linear combination of the original variables (alleles) with the largest between-group variance and the smallest within-group variance. The initial DAPC analysis was set-up to describe the maximum proportion of variance (95%). The subsequent test of a trade-off between the power of discrimination and over-fitting by calculation of the α-score resulted in ten principal components. The final DAPC analysis was conducted on ten principal components (PCs) and four discriminant functions (DFs), representing 42.3% of the total variance conserved in the dataset using the Adegenet v2.1.3 R package [35].

Unsupervised network analysis utilising the Super Paramagnetic Clustering approach [36] was carried out to derive fine-scale stratification between and among analysed groups using the Netview R package [37]. The symmetric matrix of the identity-by-descent (IBD) distances for all pairs of individuals calculated in PLINK 1.9 [29] was used as an analysis input. The maximum number of interconnected nearest neighbours (k-NN) ranged from 5 to 30, according to the k-NN selection plot. The k-NN = 15 was selected to visualise the population-wide genetic structure.

The proportion of admixture between analysed groups was estimated by the unsupervised Bayesian clustering approach implemented in Structure 2.3.4 [38]. The analysis was carried out using the default parameter of an admixture model and correlated allele frequencies based on a burn-in period of 10,000 followed by 100,000 MCMC (Markov chain Monte Carlo) replications. Ten runs were performed from K = 1 to K = 10. The optimal number of K was selected based on the probability of delta K (ΔK), according to Evanno et al. [39].

### 2.4. Population Differentiation Based on Variants Near Genes of Relevance for the Selected Breed Phenotypes

Assuming that the natural and artificial selection acting during breed formation can leave various traces in the animal genome, the genetic differences among evaluated groups were also estimated by analysing the frequency of alleles in specific regions responsible for controlling several phenotypes. The population stratification depending on four categories of phenotypic traits was studied: aerobic trainability of the organism (ATO), behaviour and motivation (BM), coat colour (CC), and strength and endurance (SE). 

The genes associated with each phenotype category were first selected by a literature survey (Table S1). As there is a lack of information about the genetic control of ATO and SE in dogs, the genome-wide association studies in humans as a model organism were used as an additional data source. The biological importance of selected genes was then tested by a web-based gene set analysis toolkit WebGestalt [40] separately for each phenotypic category. The gene ontology (GO) terms associated with *Canis lupus familiaris* gene stable IDs were retrieved via over-representation analysis (ORA). The significantly enriched categories were identified based on the Bonferonni method, which is one of the most commonly used multiple test adjustment approaches. The top ten GO significantly over-represented biological processes (*p* < 0.05) were considered. In the next step, the chromosomal position of selected genes in the dog genome was determined by the ensembldb R package [41], and all of the SNPs located out of gene-coding sequences were pruned out from the dataset. Finally, the four sub-datasets containing genetic variants associated with particular phenotypic traits were prepared. The population stratification due to a specific allelic frequency was then tested separately for each phenotype category by PCA and factor analysis (FA) in the R program [42]. In FA, each of the selected genes was considered as a separate factor potentially affecting the frequency of particular alleles.

## 3. Results and Discussion

### 3.1. Effect of Selection on the Genome-Wide ROH Distribution

**Figure 1:**
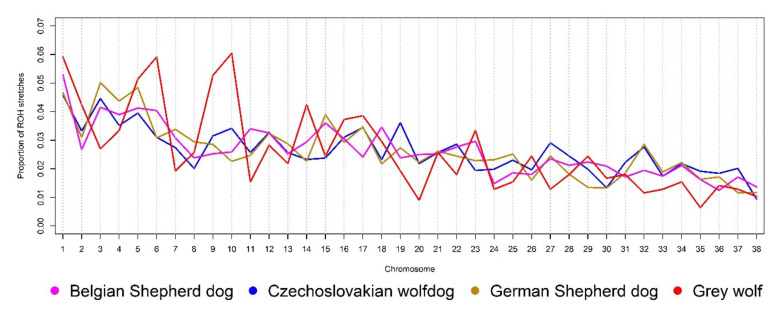
The relative proportion of ROH segments across the autosomal genome of the analysed groups.

As shown in previous studies [43,44], natural and artificial selection are the driving forces of evolution that significantly shaped the genome of dogs. By analysing high-density SNP data, it is possible to express the frequency and length of homozygous segments arising as a consequence of selection pressure on particular genomic regions, mainly controlling favourable phenotypic traits [45]. Figure 1 shows the ROH distribution across the autosomal genome of the analysed groups. Saarloos wolfdog was not included in this analysis, due to the limited number of sampled animals and their high genomic uniformity. The comparison of autosome-wide ROH proportion determines significant differences between the Grey wolf and analysed dog breeds, probably resulting from different selection events (natural and/or artificial) acting in the past. Thus, the results point to the need for a fine-scale analysis of their genomes to identify the source of the observed differences and, where appropriate, to identify breed-specific patterns inherited as part of the certain traits defined in the standards and goals of each breed. Simultaneously, it is possible to use detected ROH to express the trend of inbreeding intensity in past generations [6]. Due to well-known principles of breed formation and their history, it is necessary to distinguish between historical inbreeding resulting from the restricted number of founders and inbreeding of the current generation arising in the population due to the nonequal use of founders, leading to a limited number of common ancestors permanently appearing in the pedigrees as a result of the mating programme. Observed differences between breeds may also be related to preferential mating and breeder preference, including favouring particular sire lines and maternal families. Especially in small local populations, preferential mating can significantly affect the inbreeding intensity in the current generation [7].

Table 2 summarises the results of the ROH distribution scan in the autosomal genome of the analysed groups. The Czechoslovakian wolfdog is a relatively young breed whose origin is underlined by a restricted number of Carpathian grey wolves repeatedly used in mating and crossbreeding with German Shepherd dogs [46]. Due to this fact and the rigorous selection in the direction of the CWD breeding goal, it is obvious that the limited number of founders has a strong effect on the proportion of ROH segments within each length class. In the CWD genome, the highest proportion of very short (0–2 Mbp; 38.05%) and short (2–4 Mbp; 22.61%) ROH segments, with average lengths of 1.13 and 2.89 Mbp, respectively, were found. These ROH length classes correspond to a historical load of inbreeding derived from founders, simultaneously representing inbreeding inherited from base animals of the source populations, i.e., especially GSDs. Long ROH segments (4–8 and 8–16 Mbp) reflect the inbreeding of common ancestors of animals used in the breeding, and very long ROH segments (>16 Mbp) point to the proportion of relatives mating in the current generation [47]. The ROH > 16 Mbp are most commonly used to derive the level of genomic inbreeding in the current generation, here denoted as *F_ROH16_*. In the CWD, the average recent genomic inbreeding at a level of 7.48 ± 3.41% was found. The high standard deviation results from the high ROH length variability observed within this class (average segment length of 21.18 Mbp).

**Figure 2:**
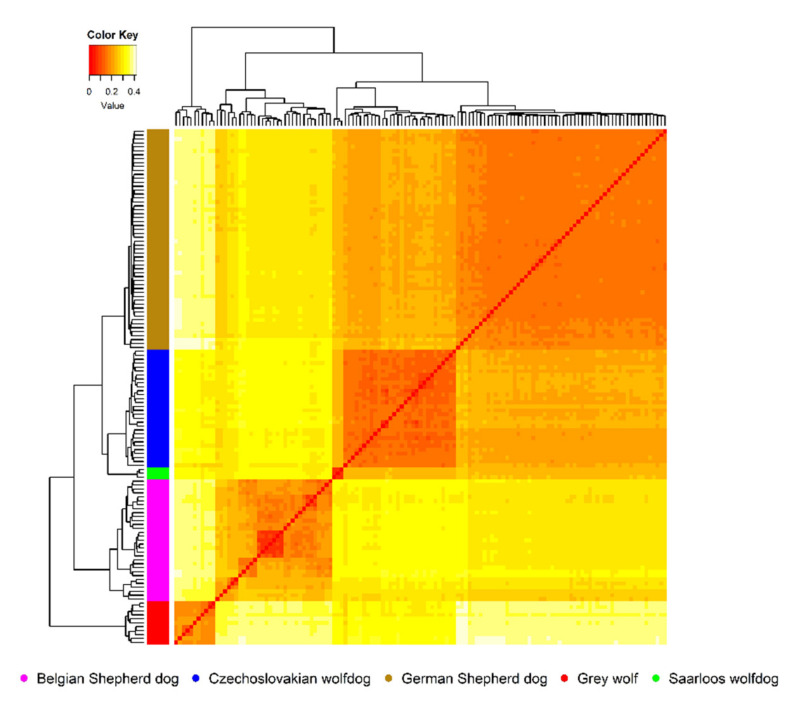
Detailed insight on an intrapopulation genetic structure derived from the Nei’s genetic distance matrix.

Compared to the GSD, the CWD showed a lower genome-wide proportion of short ROH segments and a higher proportion of long segments (Table 2), confirming that the inbreeding events shaped the CWD genome only a few generations ago, as reported by Caniglia et al. [1]. The ROH structure of the GSD genome considers the longer history of the breed and corresponds to the fact that this breed is more genomically uniform, as presented in Figure 2. The high occurrence of very short ROH segments proposes the repeated use of founders in GSD breeding and the preferential mating of selected genotypes in subsequent generations. The high genomic uniformity of GSDs is manifested in their phenotypic uniformity as well. The obtained average value of *F_ROH16_* (4.46%) in GSDs was lower than that in CWDs, mainly due to the large selection basis and worldwide distribution of the breed. The distribution of ROH within length classes in BSDs followed a similar trend as in GSDs. The difference was evident mainly in the lower number of very short ROH segments (0–2 Mbp) observed in BSDs; however, their proportion of the total number of detected ROH was similar (49.77%). GW showed a significantly lower proportion of ROH segments across the analysed length classes than the others, as well as low genomic inbreeding derived from the ROH distribution. The observed proportion of ROH within each class due to natural selection and preservation of population fitness was expected. The highest proportion of the short ROH segments (83.28%) in the GW genome may be related to the fact that those are either responsible for survival or adaptation and are, therefore, inherited in the monomorphic form to next generations. The absence of ROH > 16 Mbp confirms that the mating of close relatives is not common in wild species [48].

### 3.2. Population Structure of the Breeds

The proportion of genetic similarity among tested groups derived from the calculation of *F_ST_* is shown in Table 3. The highest genetic similarity between CWD and GSD mainly reflected the fact that the GSD was used as one of the founders in CWD formation [46]. Even if both CWD and SWD were created by crossing between GSD and GW, a relatively high level of genetic differentiation between them confirmed that both breeds are genetically distant and arose at independent times as separate breeds. The CWD showed a lower genetic distance to GSD and GW compared to SWD. Despite no historical evidence of GSD or GW in BSD grading-up [49], the estimated *F_ST_* value indicated a higher genetic similarity between CWD and BSD than between CWD and GW. BSD showed, at the same time, a higher genetic similarity to GW compared to the others. The highest genetic differentiation observed between GSD and GW points to the fact that GSD is most distant from its wild ancestor compared to the others, probably as a result of bottleneck events related to the establishment of the breed.

The Nei’s genetic distances confirmed that CWD and GSD are genetically close groups as a result of the impact of GSD on the CWD genome. The Nei’s genetic distances followed the trend observed by the *F_ST_* calculation with growing genetic distances between the analysed dog breeds and GW. The highest differentiation was found between GSD and GW, while CWD showed the highest genetic similarity to GW. Generally, both the *F_ST_* and Nei’s genetic distances between the shepherd and wolf-like dog groups reflect known information from their genealogy origin. BSD was genetically closest to GSD, logically followed by SWD due to the geographical proximity of the area of breed origin. In the SWD case, Nei’s genetic distances showed a different trend than the *F_ST_* matrix, probably due to a low number of SWD animals in the analysis. Both methods proved a similar degree of genetic differentiation between CWD and SWD or BSD.

Considering wild ancestors of analysed breeds, here represented by the GW group, average values of genetic distances (*D_a_* = 0.20 ± 0.02) or genetic differentiation (*F_ST_* = 0.33 ± 0.03) indicated a sufficient level of genetic variability and interpopulation diversity among them. This fact also confirmed the matrix of *D_a_* values calculated on the intrapopulation levels (Figure 2). Figure 2 shows a graphical illustration of the overall genetic diversity of animals covered by the analysis, where colour intensity from white to red describes an increase in the degree of genetic similarity between them. The outlines of individual groups correspond to their origin.

The preference of specific phenotypes from the long-time perspective and relative uniformity of the GSD breed resulted in a high degree of genetic fixation inside this group, i.e., analysed animals showed a similar allelic frequency across all SNP markers. A high proportion of intrapopulation genetic similarity was also observed in the CWD group, indicating preferential mating concerning certain common ancestors. However, due to the current status of the breed, such preferential mating is undesirable. The GW group, whose genome was shaped by natural selection related to survival traits for a long time [16], showed a lower intrapopulation similarity than those of CWD and GSD. Such a selection pressure was not affected by the domestication process and acted in the GW genome substantially longer compared to dog breeds [50]. Due to the low number of SWD, it was not possible to evaluate its intrapopulation diversity. The SWD group was only used to describe better interpopulation relationships within groups of breeds derived from GW. As shown in Figure 2, the intrapopulation genetic fragmentation occurred mainly inside the BSD group. The observed fragmentation is caused by the fact that this study included various BSD types, which significantly differs in their phenotypes (Malinois, Tervuren, Laekenois, and Groenendael) [51].

**Figure 3:**
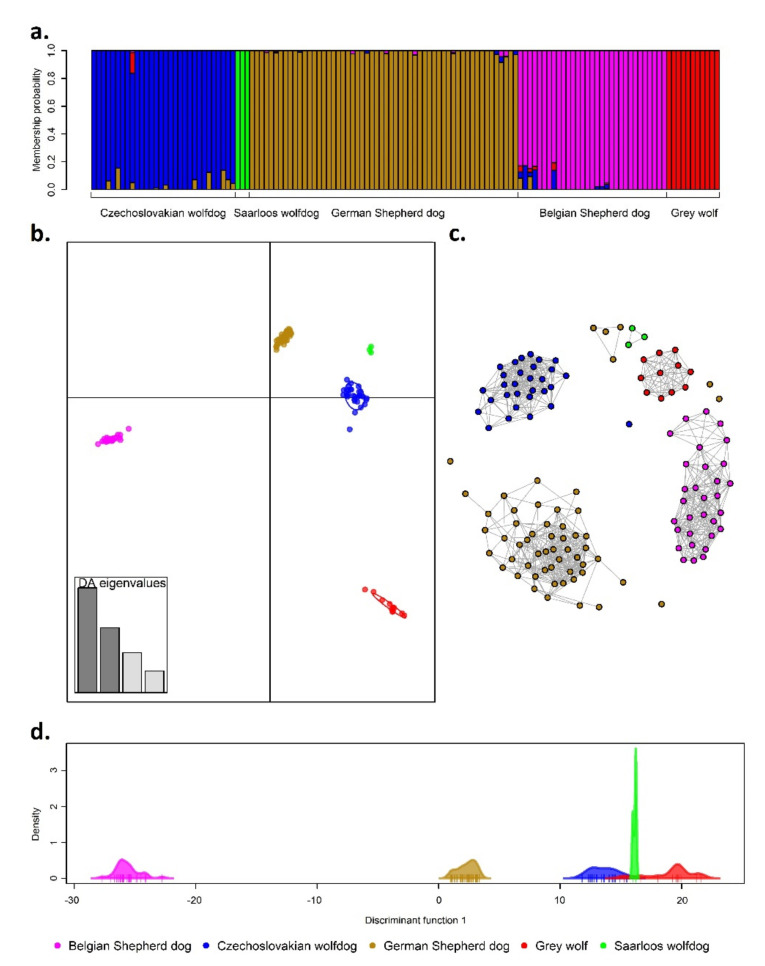
Fine-scale population structure based on stacked barplot of the cluster membership suggested by the Bayesian algorithm (**a**), first two discriminant functions of supervised DAPC analysis (**b**), the mutual nearest-neighbour graph obtained from unsupervised clustering method (**c**), and the first discriminant function of DAPC analysis (**d**).

Differences in the genetic formation of individual groups are presented in Figure 3. Each of the applied methodological approaches confirmed that the analysed groups formed genetically different units with a relatively low level of admixture between them (up to 4%), as suggested by *F_ST_* and Nei’s genetic distances. The highest proportion of admixture was found in the CWD genome (Table 4), where GSD and GW were recognized as the groups with the highest contribution to its genetic makeup. A similar level of admixture was found in the BSD genome, which showed genetic traces of CWD, GSD, and GW as well (Figure 3a). Figure 3b, presenting the first two discriminant functions of DAPC analysis, point out separate genetic clusters according to group origin. Even if there was a significant genetic similarity between wolf-like breeds (CWD and SWD) and GSD, the analyses showed that the GW group, as their wild ancestor, was genetically separated from the others. Figure 3c shows a vertical genetic structure of the analysed groups based on the first discriminant function of DAPC analysis. The first discriminant function indicated only three clusters, where the CWD and SWD groups were clustered together with GW. This can be explained by the higher proportion of wolf-like genotypes in the CWD and SWD genome compared to GSD and BSD. The fine-scale stratification between and among the analysed groups resulting from the IBD matrix (Figure 3b) confirmed that the breeds formed genetically separate clusters that can be reliably distinguished based on the genomic data.

### 3.3. Population Differentiation Based on Variants Near Genes of Relevance for the Selected Breed Phenotypes

The population stratification analysis was based on the assumption that both natural and artificial selection acting in specific genomic regions during breed development can leave significant traces in the genome detectable at the allelic frequency level. The differences in allelic frequency among the analysed groups were tested depending on the four categories of phenotypic traits (aerobic trainability of the organism, behaviour and motivation, coat colour, and strength and endurance) that form an important part of breed standards and breeding goals. Even if the analysed breeds were selected for very similar purposes, there are several significant differences in their breeding goals. Both GSD and BSD are recognized as multipurpose working dogs, while in CWD and SWD, wolf-like phenotypes are predominant in selection (e.g., coat colour). In addition, the CWD is the only breed where the endurance is directly included in the breeding goal in the form of the first-degree exam consisting of a 40 km endurance run.

**Figure 4:**
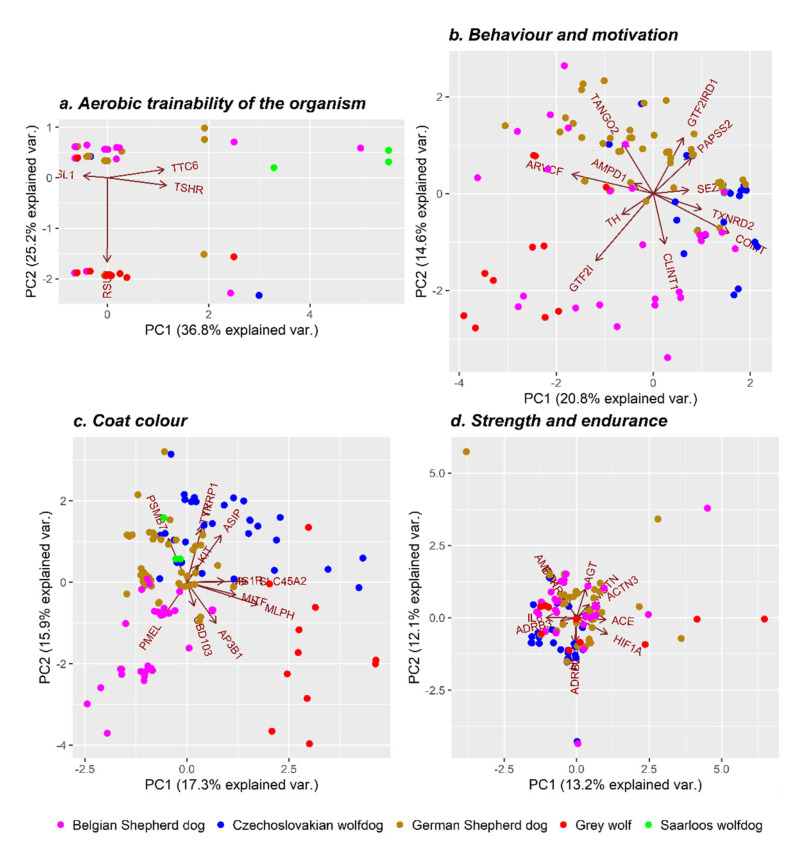
Population differentiation based on variants near genes of relevance for the selected phenotypic traits (aerobic trainability of the organism (**a**), behaviour and motivation (**b**), coat colour (**c**), and strength and endurance (**d**)).

A literature survey indicated four genes (*RSU1*, *TTC6*, *TSHR*, and *ACSL1*) that can potentially affect the organism aerobic trainability through control of the expression of oxidative capacity (VO_2Max_) (Table S1). The gene ontology (GO) term analysis of the biological function of selected genes resulted in various significantly enriched GO terms mostly related to *RSU1* and *TSHR* genes (Table S2). In humans, the *RSU1* gene is responsible for the proper development and differentiation of muscle mass and interaction with multiple signalling pathways, including positive regulation of the catalytic activity, molecular function, and cell–substrate adhesion [52]. On the other hand, the *TSHR* gene serves as a membrane receptor for thyrotrophic, a major controller of thyroid cell metabolism. Thyroid hormones are considered determinants of the metabolic and contractile phenotype of skeletal muscles [53]. As shown in Figure 4a, the diversity analysis based on the genetic polymorphism of the tested genes connected to aerobic trainability showed mainly individual differences, which could not be attributed to individual breeds. Only animals representing the SWD group are slightly vector-distributed due to the effect of *TTC6* gene polymorphism in humans associated with the VO_2Max_ training response [54]. In general, it can be, therefore, assumed that during breed development, the emphasis was on explosive power and anaerobic endurance; therefore, alleles (haplotypes) responsible for these traits remained equally distributed in the genome of the analysed dog breeds, as well as their wild ancestor.

The behaviour and coat colour of dogs belong to the phenotypic traits where the intense selection pressure significantly acted for a long time. In the case of behaviour and motivation, a set of 11 genes was selected to assess the level of genetic diversity distributed within the analysed animals (Table S1). The GO analysis (Table S3) indicated as significantly enriched mainly the GO terms comprising *TH*, *COMT*, and *SEZ6L* genes that have, in previous studies, been associated with several traits in humans and dogs. Tyrosine hydroxylase (*TH*) is involved in synthesising the dopamine precursor, which acts as a precursor of the catecholamines noradrenaline and adrenaline. Except for the brain’s reward system, *TH* is responsible for various important biological functions in organisms (Table S3), including control of movement, cognition, and attention [55]. The *COMT* gene is associated with many metabolic processes, including the neurotransmitter metabolic process, the regulation of neurotransmitter levels, and executive function. Concerning executive function, sensitivity to reward may be a key factor in cognitive task performance [56]. The *SEZ6L* gene has been previously associated with human autism, a neurodevelopmental disorder connected to impairment in social interactions and communication. In dogs, both *COMT* and *SEZ6L* genes were linked to social behaviour in Golden retrievers and Labradors [24,27]. The highest observed differences in genetic variability between the Grey wolf and the domesticated dog breeds were manifested by the SNPs’ allelic frequency inside the *GTF21* and *GRF2IRD1* genes (Figure 4b), which divided the analysed groups. Both genes were linked to the level of social context-dependent salivary oxytocin [25]. Therefore, the observed population stratification points to the differences in oxytocin production in the owner’s presence, which genetically classify domesticated breeds into a different cluster compared to their wild ancestor. In addition, the CWD and SWD were partly differentiated from others due to the frequency of SNPs inside genes associated with social behaviour disorders (*SEZ6L*, *COMT*, *TXNRD2*). In CWD, such social behaviour disorders are mainly correlated with maternal instinct (e.g., litter protection, hiding, and shyness).

The population stratification due to coat colour variation among groups was tested based on the set of SNPs distributed within sequences of 12 protein-coding genes (Table S1). Table S4 shows the 10 most important biological pathways resulting from the GO terms enrichment analysis. The subsequent analysis of genetic differences between the tested groups showed four partly separated clusters. The GW group differs from other domesticated breeds mainly due to the effect of *MLPH*, *MITF*, and *AP3B1* genes. The melanophilin gene (*MLPH*) is a candidate gene responsible for eumelanin dilution. In animals with the black coat colour, a mutation in the *MLPH* gene leads to the transition from a grey to a blue coat colour, while for a red colour, there is a transition to a cream colour [57]. The microphthalmia-associated transcription factor gene (*MITF*) is involved in the genetic control of the white-spotted coat colour [58]. The *AP3B1* gene polymorphism was previously associated with canine cyclic neutropenia, known as Collie syndrome, causing a diluted coat colour [59]. The BSD group showed differences in the allelic frequency of premelanosome protein (*PMEL* or *SILV*) and β-defensin 103 (*CBD103*) genes. The *PMEL* gene affects the so-called merle colour, which is characterized by spots of dilute pigment mixed with normal melanin. Blue eyes often occur in individuals who have this gene. The merle coat colour is autosomally inherited with incomplete dominance. Dominant homozygotes are completely white, heterozygotes have a merle coat colour, and recessive homozygotes have a normal colour [60]. The *CBD103* gene controls pigment type by competitively binding to the *MC1R* gene and switching responsible pathways. The *CBD103* gene in the dominant form leads to the black coat colour [61]. The CWD group was genetically separated from the others mainly due to the frequency of SNPs inside *KIT, ASIP, TYR, TYRP1, MC1R*, and *SLC45A2* genes. In dogs, melanocortin receptor 1 (*MC1R*) and agouti-signalling protein precursor (*ASIP*) genes are responsible for the genetic control of melanocytes, producing pheomelanin (red or yellow pigment) or eumelanin (black or brown pigment). The *MC1R* gene acts epistatically to *ASIP* and *CBD103* genes [61]. Activated *MC1R* gene-producing pheomelanin results in a uniform black coat colour, whereas the inhibited form is responsible for the red or yellow colour due to the production of pheomelanin [62]. Mutations in the *MC1R* gene were also linked to the black mask pattern [63]. The *ASIP*, similar to the *CBD103* gene, controls pigment type. Gain-of-function mutations of the *ASIP* gene lead to the dominant inheritance of the yellow colour, while loss-of-function mutations result in recessive inheritance of the black colour [61]. Mutations in the *ASIP* gene can result in a saddle tan or black-and-tan coat colour as well [13]. The tyrosine-related protein 1 gene (*TYRP1*) encodes an enzyme present in the synthesis of eumelanin and affects the brown coat colour [64]. The *TYRP1* gene interacts with the *MC1R* gene. The *TYRP1* dominant allele can only be expressed if the individual simultaneously has the *EE* or *Ee* genotype of the *MC1R* gene [65]. The tyrosinase (*TYR*) and solute carrier family 45, member 2 (*SLC45A2*) genes belong to the group of novel identified genes that are often associated with albinism in dogs [66]. The KIT gene was identified as the gene causing the so-called white spotting in several mammalian species. The white areas on the coat are depigmented due to the absence of pigment-producing melanocytes. Mutations of this gene were also found in the German Shepherd [67]. In the middle of the analysed groups, a cluster composed of SWD and GSD (Figure 4c) was located. Even if there was a strong selection pressure for the fixation of alleles controlling the wolf-like coat texture, colour, and mask in SWD, the obtained results showed that the animals carry different allelic combinations than GW does. 

Strength and endurance are antagonistic traits, whose expression is encoded by various genes. For this study, a set of 11 genes was selected based on previous research (Table S1) [. As shown by the GO enrichment analysis, some of the selected genes were simultaneously included in several identified GO terms (*AGT*, *ADRB2*, *ADRB3*, *HIF1A*, *BDKRB2*, and *MSTN*) (Table S5). It was reported that blood pressure regulation associated with the *AGT* gene is essential for muscle functions [68]. The β-2 and β-3 adrenergic receptors (*ADRB2* and *ADRB3*) genes control energy expenditure and lipolysis and are important for cardiovascular system optimisation during muscle activity. Both genes were associated with elite endurance performance in humans [69,70,71]. Hypoxia-inducible factor 1 (*HIF1*) is a transcription factor that regulates gene expression in response to hypoxia that has been associated with athletic performance [72]. Variation in the bradykinin B_2_ receptor (*BDKRB2*) implicated in the increase in skeletal muscle glucose uptake during exercise has been associated with endurance performance [73]. The *MSTN* gene, as a member of the transforming growth factor β family, encodes the myostatin protein. The *MSTN* controls the total number of muscle fibres by regulating overall myoblast proliferation [74]. As can be seen in Figure 4d, the GW and CWD partly differentiated from other groups due to variations in *ADRB2*, *ADRB3*, and *IL6* genes, which play important roles in the regulation of the cardiovascular system, vasodilation, and efficiency of access to fat stores. Interleukin 6 (*IL6*) modulates the release of different cytokines, such as TNF and IL1Ra. Interleukin 6 plasma concentration is affected by exercise duration, intensity, and the amount of muscle mass involved [75]. Table S1 cited the references [17,24,25,27,54,58,63,66,67,69,71,72,73,74,76,77,78,79,80,81,82,83,84,85,86,87,88].

## 4. Conclusions

Historical selection events have left different traces in the genomes of the analysed groups that have differentiated them from each other. In the Czechoslovakian wolfdog, a strong effect of rigorous selection in the direction of its breeding goal was manifested mainly in a high genome-wide proportion of short ROH segments, corresponding to a historical load of inbreeding inherited from base animals of the source populations. The observed proportion of long ROH segments confirmed that the inbreeding events shaped the Czechoslovakian wolfdog genome relatively recently compared to the other analysed groups. In Grey wolf, a considerably lower genome-wide proportion of ROH segments compared to domesticated breeds was found mainly as a consequence of natural selection effects related to population fitness preservation. The absence of long ROH segments in the Grey wolf genome follows from the fact that the mating of close relatives is not common in wild species. Due to the sufficient level of interpopulation genetic variability, each of the analysed groups formed a separate genetic cluster according to their origin. As expected, the highest proportion of admixture was found in the Czechoslovakian wolfdog genome, where the German Shepherd dog and Grey wolf were recognized as the groups with the highest contribution to its genetic makeup. Even if there was a significant increase in genetic connectedness among wolf-like breeds and their wild ancestor due to the strong selection pressure for the fixation of alleles controlling the so-called wolf-like phenotype, the obtained results showed that the Czechoslovakian wolfdog carries different allelic combinations that are not found in either wolves or other dog breeds analysed in this study.

## Figures and Tables

**Table 1:** Source of genome-wide data and sample size of analysed breeds.

Breed	Abbreviation	Region of Origin	Sample Size	Genotyping Platform	Data Source
Belgian Shepherd dog	BSD	Belgium	31	CanineHD 170k	Shannon et al. [30]
Czechoslovakian wolfdog	CWD	Former Czechoslovakia	30	CanineHD 230k	This study
German Shepherd dog	GSD	Germany	56	CanineHD 170k	Shannon et al. [30]
Grey wolf	GW	Eurasia	30	CanineHD 170k	Shannon et al. [30]
Saarloos wolfdog	SWD	Netherlands	3	CanineHD 230k	This study

**Table 2:** Summary statistics for detected ROH per breed and ROH class.

Breed	ROH Class (Mbp)	No. of ROH (Mean Length in Mbp)	Distribution of ROH (%)	*F_ROH_* ± SD
CWD ^1^	0–2	1868 (1.129)	38.053	35.010 ± 4.870
	2–4	1110 (2.893)	22.612	31.818 ± 4.694
	4–8	1088 (5.704)	22.163	26.958 ± 4.516
	8–16	616 (10.817)	12.548	17.565 ± 4.290
	>16	227 (21.771)	4.624	7.480 ± 3.406
GSD ^2^	0–2	6071 (1.146)	52.418	31.141 ± 6.572
	2–4	2669 (2.853)	23.044	25.500 ± 7.298
	4–8	1787 (5.611)	15.429	19.327 ± 7.552
	8–16	844 (10.777)	7.287	11.611 ± 6.717
	>16	211 (22.334)	1.822	4.458 ± 4.842
BSD ^3^	0–2	2231 (1.090)	49.766	24.857 ± 7.141
	2–4	975 (2.854)	21.749	21.295 ± 6.894
	4–8	735 (5.690)	16.395	17.219 ± 6.425
	8–16	405 (10.966)	9.034	11.094 ± 5.324
	>16	137 (22.869)	3.056	4.905 ± 3.534
GW ^4^	0–2	1205 (1.040)	83.276	8.508 ± 6.587
	2–4	192 (2.685)	13.269	4.077 ± 3.632
	4–8	44 (5.323)	3.041	1.898 ± 2.610
	8–16	6 (9.727)	0.415	1.325 ± 1.357
	>16	-	-	-

^1^ Czechoslovakian wolfdog, ^2^ German Shepherd dog, ^3^ Belgian Shepherd dog, ^4^ Grey wolf.

**Table 3:** Genetic distance matrix among breeds analysed based on Wright’s *F_ST_* index (under diagonal) and Nei’s genetic distances (above diagonal).

	CWD	SWD	GSD	BSD	GW
CWD		0.151	0.077	0.148	0.169
SWD	0.290		0.144	0.194	0.214
GSD	0.179	0.258		0.119	0.215
BSD	0.273	0.284	0.229		0.189
GW	0.319	0.346	0.357	0.294	

**Table 4:** The estimates of admixture proportion within breeds.

	GSD	SWD	CWD	GW	BSD
CWD	0.0251	0.0000	0.9695	0.0050	0.0005
SWD	0.0000	1.0000	0.0000	0.0000	0.0000
GSD	0.9937	0.0000	0.0027	0.0000	0.0036
BSD	0.0058	0.0000	0.0193	0.0052	0.9697
GW	0.0000	0.0000	0.0000	1.0000	0.0000

## Data Availability

Derived data supporting this study are available from the corresponding author on request.

## Supplementary Materials

The following are available online at https://www.mdpi.com/article/10.3390/genes12060832/s1, Table S1: Genes potentially associated with coat colour, performance, and behaviour traits in dogs, Table S2: The most important biological pathways associated with organism aerobic trainability based on enrichment analysis, Table S3: The most important biological pathways associated with behaviour and motivation based on enrichment analysis, Table S4: The most important biological pathways associated with coat colour based on enrichment analysis, Table S5: The most important biological pathways associated with strength and endurance based on enrichment analysis.
