# Supplementary material for: Czechoslovakian Wolfdog Genomic Divergence from Its Ancestors Canis lupus, German Shepherd Dog, and Different Sheepdogs of European Origin
Source: Genes (Basel). 2021 May 28;12(6):832. doi: 10.3390/genes12060832 (PMC8228135; doi:10.3390/genes12060832)
Supplement: Supplementary file 1 [file genes-12-00832-s001.zip › genes-1177835-supplementary.pdf]

Table S1: Genes potentially associated with coat colour, performance, and behaviour traits in dogs.

Trait	Gene symbol	Chr	Start position (bp)	End position (bp)	No. of SNPs in region	Literature source
ATO[1]	*RSU1*	2	20050117	20299184	13	Šimonek and Židek [76]
	TTC6	8	16108988	16282207	11	Williams et al. [77]
	TSHR	8	53326655	53482898	9	Ahmetov et al. [78]
	ACSL1	16	45735703	45804970	6	Bouchard et al. [54]
BM[2]	*CLINT1*	4	52447382	52505253	3	Ilska et al. [79]
	GTF2I	6	5727665	5811965	8	vonHoldt et al. [25]
	GTF2IRD1	6	5886931	5963873	7	vonHoldt et al. [25]
	AMPD1	17	52377402	52399520	2	Tsianos et al. [73]
	TH	18	46327137	46335602	2	Ilska et al. [79]
	SEZ6L	26	19888319	20076021	17	Persson et al. [24]
	TANGO2	26	29266256	29304164	3	Persson et al. [27]
	ARVCF	26	29307351	29359245	3	Persson et al. [24]
	COMT	26	29360356	29366008	2	Luo et al. [80]
	TXNRD2	26	29381975	29430206	2	Persson et al. [27]
	PAPSS2	26	37725616	37743928	1	Bouchard et al. [81]
CC[3]	*AP3B1*	3	28481038	28751258	17	Jung et al. [82]
	SLC45A2	4	73815291	73867437	3	Wijesena and Schmutz [66]
	MC1R	5	63694296	63695249	1	Schmutz and Berryere [83]
	PSMB7	9	58529723	58593016	4	Clark et al. [84]
	PMEL	10	292597	300098	2	Clark et al. [84]
	TYRP1	11	33317121	33335498	1	Schmutz et al. [63]
	KIT	13	47108504	47190029	8	Wong et al. [67]
	CBD103	16	58965216	58967712	2	Kerns et al. [85]
	MITF	20	21612927	21870578	15	Rothschild et al. [58]
	TYR	21	10799940	10894191	10	Schmutz and Berryere [83]
	ASIP	24	23354888	23393896	4	Kerns et al. [17]
	MLPH	25	48121499	48170710	3	Schmutz and Berryere [83]
SE[4]	*AGT*	4	8683798	8693880	1	Zarębska et al. [86]
	ADRB2	4	59941517	60050298	10	Wolfarth et al. [69]
	HIF1A	8	36614045	36656692	3	Eynon et al. [72]
	BDKRB2	8	64974997	65003407	1	Tsianos et al. [73]
	ACE	9	11497182	11516358	2	Huson et al. [74]
	IL6	14	36473394	36478519	2	Wallberg et al. [87]
	ADRB3	16	27445601	27447521	2	Santiago et al. [71]
	AMPD1	17	52377402	52399520	2	Tsianos et al. [73]
	ACTN3	18	50787353	50801197	2	Pimenta et al. [88]
	HFE	35	24031387	24042413	1	Semenova et al. [89]
	MSTN	37	729172	734362	2	Huson et al. [74]

[1]aerobic trainability of the organism, [2]behaviour and motivation, [3]coat colour, [4]strength and endurance

Table S2: The most important biological pathways associated with organism aerobic trainability based on enrichment analysis.

GO term	Description	Enrichment Ratio	P-value	Gene
GO:0007190	activation of adenylate cyclase activity	534.2143	0.001871	*TSHR*
GO:0045761	regulation of adenylate cyclase activity	415.5	0.002405	*TSHR*
GO:0031281	positive regulation of cyclase activity	373.95	0.002673	*TSHR*
GO:0051349	positive regulation of lyase activity	287.6538	0.003474	*TSHR*
GO:0031279	regulation of cyclase activity	249.3	0.004007	*TSHR*
GO:0043085	positive regulation of catalytic activity	14.2729	0.0049	*RSU1, TSHR*
GO:0051339	regulation of lyase activity	196.8158	0.005075	*TSHR*
GO:0044093	positive regulation of molecular function	11.08	0.008135	*RSU1, TSHR*
GO:0010811	positive regulation of cell-substrate adhesion	76.31633	0.013061	*RSU1*
GO:0050790	regulation of catalytic activity	8.328508	0.014403	*RSU1, TSHR*

Table S3: The most important biological pathways associated with behaviour and motivation based on enrichment analysis.

GO term	Description	Enrichment Ratio	P-value	Gene
GO:0042417	dopamine metabolic process	175.9765	4.84E-05	*TH, COMT*
GO:0006584	catecholamine metabolic process	142.4571	7.47E-05	*TH, COMT*
GO:0009712	catechol-containing compound metabolic process	142.4571	7.47E-05	*TH, COMT*
GO:0018958	phenol-containing compound metabolic process	67.99091	3.35E-04	*TH, COMT*
GO:0042133	neurotransmitter metabolic process	61.05306	4.15E-04	*TH, COMT*
GO:0097164	ammonium ion metabolic process	43.35652	8.24E-04	*TH, COMT*
GO:0001505	regulation of neurotransmitter levels	26.95135	0.00212	*TH, COMT*
GO:1901615	organic hydroxy compound metabolic process	15.26327	0.006486	*TH, COMT*
GO:0045471	response to ethanol	106.8429	0.009327	*TH*
GO:0070528	protein kinase C signalling	106.8429	0.009327	*SEZ6L*

Table S4: The most important biological pathways associated with coat colour based on enrichment analysis.

GO term	Description	Enrichment Ratio	P-value	Gene
GO:0043473	pigmentation	1.77E-11	6.61E-08	*AP3B1, TYRP1, KIT, TYR, ASIP*
GO:0044550	secondary metabolite biosynthetic process	6.30E-08	1.18E-04	*TYRP1, TYR, ASIP*
GO:0033059	cellular pigmentation	2.33E-07	2.44E-04	*AP3B1, TYRP1, ASIP*
GO:0046189	phenol-containing compound biosynthetic process	2.77E-07	2.44E-04	*TYRP1, TYR, ASIP*
GO:0019748	secondary metabolic process	3.25E-07	2.44E-04	*TYRP1, TYR, ASIP*
GO:0046148	pigment biosynthetic process	5.05E-07	3.15E-04	*TYRP1, TYR, ASIP*
GO:0042440	pigment metabolic process	9.33E-07	4.99E-04	*TYRP1, TYR, ASIP*
GO:0018958	phenol-containing compound metabolic process	3.75E-06	0.001756	*TYRP1, TYR, ASIP*
GO:1901617	organic hydroxy compound biosynthetic process	3.62E-05	0.015071	*TYRP1, TYR, ASIP*
GO:0030318	melanocyte differentiation	4.17E-05	0.015599	*TYRP1, KIT*

Table S5: The most important biological pathways associated with strength and endurance based on enrichment analysis.

GO term	Description	Enrichment Ratio	P-value	Gene
GO:0097755	positive regulation of blood vessel diameter	175.2891	4.47E-07	*ADRB2, BDKRB2, ADRB3*
GO:0019220	regulation of phosphate metabolic process	9.063885	5.58E-07	*AGT, ADRB2, HIF1A, BDKRB2, ADRB3, MSTN*
GO:0051174	regulation of phosphorus metabolic process	9.063885	5.58E-07	*AGT, ADRB2, HIF1A, BDKRB2, ADRB3, MSTN*
GO:0003044	regulation of systemic arterial blood pressure mediated by a chemical signal	140.2313	9.08E-07	*AGT, ADRB2, ADRB3*
GO:0003073	regulation of systemic arterial blood pressure	77.90625	5.64E-06	*AGT, ADRB2, ADRB3*
GO:0042325	regulation of phosphorylation	8.80573	9.00E-06	*AGT, ADRB2, HIF1A, BDKRB2, ADRB3, MSTN*
GO:1902531	regulation of intracellular signal transduction	8.094156	1.47E-05	*AGT, ADRB2, HIF1A, BDKRB2, ADRB3, MSTN*
GO:0035296	regulation of tube diameter	56.0925	1.54E-05	*ADRB2, BDKRB2, ADRB3*
GO:0097746	regulation of blood vessel diameter	56.0925	1.54E-05	*ADRB2, BDKRB2, ADRB3*
GO:0009966	regulation of signal transduction	5.490038	1.77E-05	*AGT, ADRB2, HIF1A, BDKRB2, IL6, ADRB3, MSTN*
